# Subtyping of microsatellite instability-high colorectal cancer

**DOI:** 10.1186/s12964-019-0397-4

**Published:** 2019-07-22

**Authors:** Wangxiong Hu, Yanmei Yang, Lina Qi, Jiani Chen, Weiting Ge, Shu Zheng

**Affiliations:** 1 Cancer Institute (Key Laboratory of Cancer Prevention and Intervention, China National Ministry of Education), The Second Affiliated Hospital, Zhejiang University School of Medicine, Zhejiang, 310009 Hangzhou China; 2 Key Laboratory of Reproductive and Genetics, Ministry of Education, Women’s Hospital, Zhejiang University School of Medicine, Zhejiang, 310006 Hangzhou China

**Keywords:** Colorectal cancer, Microsatellite instability-high, Subtyping, Tumor-associated macrophages, Tumor-infiltrating lymphocytes

## Abstract

**Background:**

Patients with microsatellite instability-high (MSI-H) colorectal cancer (CRC) generally have a better prognosis than patients with microsatellite stable (MSS) CRC. However, some MSI-H CRC patients do not gain overall survival benefits from immune checkpoint-blockade treatment. In other words, heterogeneity within the subgroup of MSI-H tumors remains poorly understood. Thus, an in-depth molecular characterization of MSI-H tumors is urgently required.

**Methods:**

Here, we use nonnegative matrix factorization (NMF)-based consensus clustering to define CRC MSI-H subtypes in The Cancer Genome Atlas and a French multicenter cohort GSE39582. CIBERSORT was used to calculate the proportions of 22 lymphocytes in tumor tissue in MSI-H subtypes.

**Results:**

MSI-H CRC samples basically clustered into two subgroups (MSI-H1 and MSI-H2). MSI-H1 was characterized by a lower *BRAF* mutational status, higher frequency of chromosomal instability, global hypomethylation, and worse survival than MSI-H2. Further examination of the immune landscape showed that macrophages of the M2 phenotype were enriched in MSI-H1, which may be associated with poor prognosis in this subgroup.

**Conclusions:**

Our results illustrate the genetic heterogeneity in MSI-H CRCs and macrophages may serve as good targets for anticancer therapy in MSI-H1.

**Graphical abstract:**

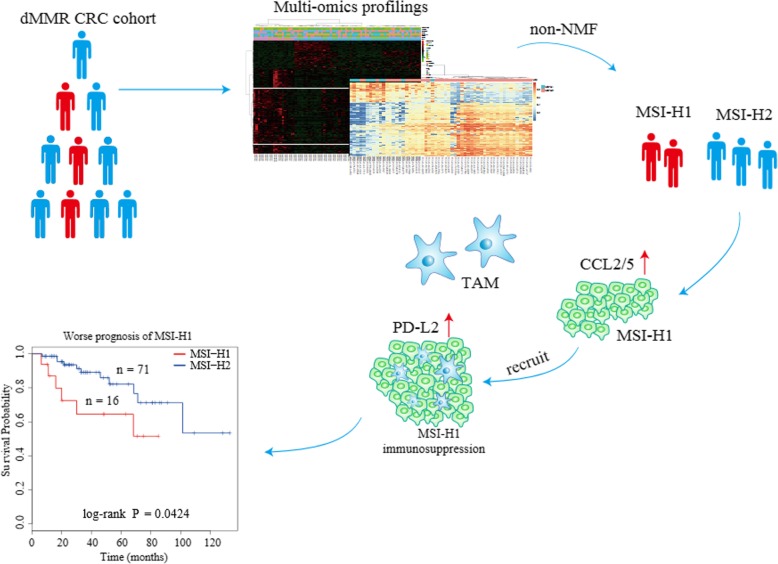

**Electronic supplementary material:**

The online version of this article (10.1186/s12964-019-0397-4) contains supplementary material, which is available to authorized users.

## Background

Much effort has been devoted to the molecular subtyping of colorectal cancer (CRC) based on gene expression profiles [1–3]. According to the current widely accepted consensus molecular subtype (CMS) classification system, microsatellite instability-high (MSI-H) samples belong to the CMS1 subtype and are characterized by hypermutation and CpG island methylator phenotype (CIMP). The MSI-H status (commonly linked to a high mutational burden) may be associated with a better prognosis due to the accumulation of somatic mutations [4, 5]. However, we previously found that even in a hypermutated subpopulation can be further divided into two groups with either a high or low prognostic index [6]. In addition, MSI-H tumors generally respond well to immunotherapy by anti-PD-1 immune checkpoint inhibition [7]. Nevertheless, *JAK1* loss-of-function mutations, with a prevalence of 20% in MSI-positive CRC are associated with the upregulation of genes associated with resistance to anti-PD-1 treatment [8]. Thus, the MSI-H population may also display different expression patterns that are masked by higher variations relative to other subtypes, such as CMS2–4.

In this study, we used nonnegative matrix factorization (NMF)-based consensus clustering to define MSI-H CRC subtypes. Intriguingly, we found that MSI-H CRC can be separated into two different subtypes with distinct molecular profiles, which help us better understand the heterogeneity within MSI-H.

## Materials and methods

### Somatic mutation data retrieval and processing

CRC somatic mutation data and clinical information were downloaded from The Cancer Genome Atlas (TCGA) data portal (05/03/2015). Silent mutations, RNA mutations, and any mutation in the intron, 5′ untranslated region (UTR), and 3’UTR were discarded. Retained mutational profiles of each case were used to refine the list of mutated genes in a total of 59 MSI-H tumors [9]. Lastly, clinical information of each patient (Additional file 1: Table S1) was added to mutational information via unique patient ID.

### Gene expression data processing and normalization

Level 3 tumor mRNA expression data sets (RNASeqV2) were obtained from TCGA (October 2015). The GSE39582 (Affymetrix HG U133 Plus 2.0 arrays) dataset was downloaded from the Gene Expression Omnibus (GEO). Raw CEL files were processed using the *affy* package of BioConductor [10]. Then, MAS5 algorithm was used for background correction, normalization and summarization of single probes for all probe sets. Analysis of differentially expressed mRNA was performed using the *DEGSeq* package for R/Bioconductor [11]. Genes with expression levels < 1 (RNA-Seq by Expectation Maximization (RSEM)-normalized counts) in more than 50% of samples were removed. Significant differentially expressed mRNAs were selected according to a false discovery rate (FDR)-adjusted *P* value < 0.05 and fold change > 2 conditions. NMF was performed using the *NMF* package for R [12].

### HM450k data retrieval and processing

DNA methylation data (HumanMethylation450) were downloaded from TCGA data portal (11/13/2016). The methylation level of each probe was measured with a beta value ranging from 0 to 1; this value is calculated as the ratio of the methylated signal to the sum of the methylated and unmethylated signals. Probes with an “NA” value in more than 10% of the CRC samples were discarded. Next, the Bioconductor package *limma* was used to identify differentially methylated sites (DMSs) in the remaining probes [13]. Significant DMSs were selected according to FDR-adjusted *P* value < 0.01 and beta value change > 0.2. All heatmaps were generated using the *pheatmap* package in R (64-bit, version 3.0.2).

### Gene ontology (GO) and Kyoto encyclopedia of genes and genomes (KEGG) enrichment analyses

GO and KEGG enrichment analyses were performed using the *clusterProfiler* package from BioConductor [14]. Significantly enriched GO terms and pathways were selected according to an FDR-adjusted *P* value < 0.01.

### Network construction and hub gene definition

Coexpression network construction was performed as described in our previous work [15]. Hub genes were those with an extremely high level of connectivity in a given network. Connectivity reflects how frequently a node interacts with other nodes and the sum of the weights across all edges of a node. Here, the top 50 genes with the highest connectivity in each module that were reasonable to display were defined as hub genes as previously described [16].

### Survival analysis

Survival differences between MSI-H1 and MSI-H2 were tested by the Kaplan-Meier method and analyzed with the log-rank test with functions *survfit* and *survdiff* in the *survival* package for R [17]. Cox univariate model was carried out with function *coxph* in the R package *survival*. A *P* value < 0.05 was considered significant.

### Deciphering lymphocytes in tumor tissue in MSI-H populations

To accurately quantify the relative amount of distinct lymphocytes in tumor tissue, CIBERSORT was used to calculate the proportions of 22 lymphocytes in tumor tissue [18]. The permutations were set to > = 100, and quantile normalization (QN) of the input expression mixture was set to FALSE for TCGA RNAseq data.

### Immunohistochemistry (IHC) of Zhejiang University cancer institute (ZUCI) dMMR samples

Subtyping of 28 ZUCI dMMR frozen tissue samples was based on RNA-seq dataset. Matched formalin fixed paraffin-embedded (FFPE) samples were collected from pathology department. IHC staining and semi-quantitative analysis were performed as our previous work [19]. The four μm sections were incubated with the anti-CD68 (1:500 dilution, Cell Signaling Technology, 76437) and CD163 (1:500 dilution, Cell Signaling Technology, 93498) antibody, respectively.

## Results

### MSI-H CRC clusters into two distinct subtypes

**Fig. 1:**
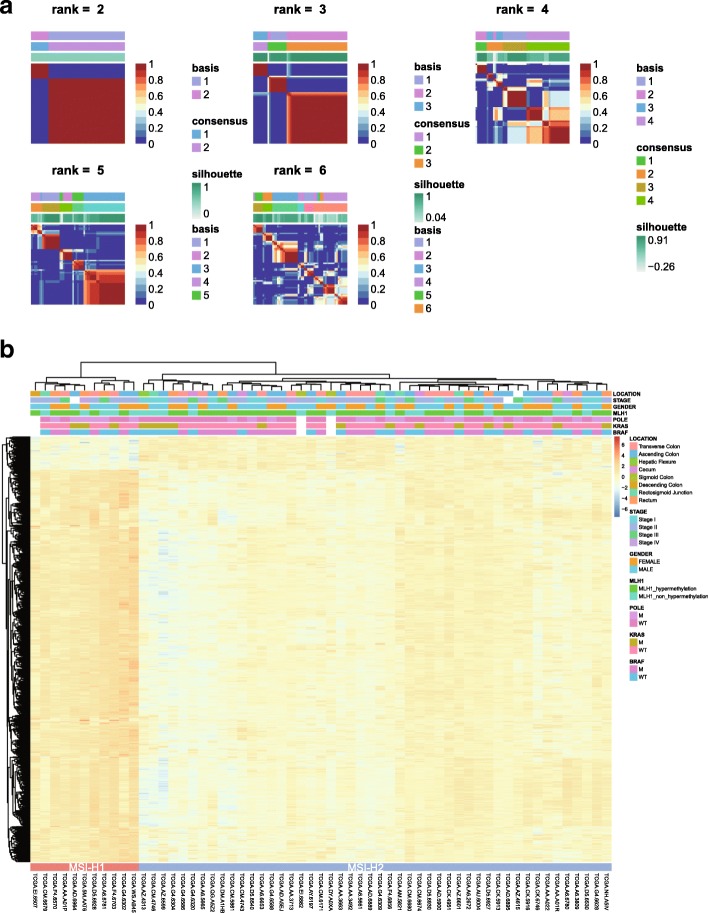
MSI-H CRC cases clustered into two gene expression-based subtypes. **a**, Clustering of 59 MSI-H CRC cases in TCGA by NMF. Correlation matrix heatmaps correspond to ranks 2 to 6. **b**, The association of clinicopathologic factors and MSI-H subtypes is shown on the top. Heatmap displaying the expression pattern of MSI-H subtypes

A stochastic NMF algorithm was used to assess whether any clusters were present in the MSI-H CRC cases. To determine the best factorization rank *r*, a critical parameter in NMF, different values two to six was calculated. Then, the best *r* value was chosen according to some quality measures, such as the first value of *r* for which the cophenetic coefficient starts decreasing, the first value where the residual sum of squares (RSS) curve presents an inflection point [20], and direct visual inspection of the consensus matrix. Notably, *r* = 2 met all of these quality criteria, in other words; the MSI-H tumors fell into two separate subgroups (Fig. 1a). Of the 59 MSI-H samples annotated in TCGA, 11 were classified into MSI-H group 1 (MSI-H1) and 48 were classified into MSI-H group 2 (MSI-H2). The ratio of MSI-H1 to MSI-H2 was approximately 1:4 (Fig. 1b).

To validate the MSI-H subtypes found in TCGA dataset, we used the GSE39582 dataset [21] with 77 dMMR (deficient in DNA mismatch repair system (MMR)) samples to explore clustering (Additional file 2: Figure S1). Consistent with the two subtypes of MSI-H CRC in TCGA, two clusters were also observed in the French multicenter cohort (MSI-H1: MSI-H2 was approximately 1:3, Additional file 3: Figure S2). MSI-H CRC has a relatively good prognosis when diagnosed as stage II and a poor prognosis when diagnosed as advanced stages, suggesting that stage IV MSI-H CRC may differ considerably at the molecular level from stage II MSI-H CRC. As stage II CRC has the highest prevalence of MSI (81% in TCGA and 69% in GSE39582), a separate MSI subtyping was performed for stage II CRC only. Results showed that MSI subtyping remained basically unchanged (MSI-H1 and MSI-H2), suggesting that MSI-H CRC molecular subtyping was independent of tumor stages (Additional file 4: Figure S3 and Additional file 5: Figure S4).

### MSI-H1 harbors a lower *BRAF* mutational frequency and a higher frequency of chromosomal instability

The ratio of MSI-H1: MSI-H2 closely corresponds to Lynch syndrome-related tumors and sporadic MSI tumors, which are two major classes of CRC. To test this hypothesis, we extracted the methylation probes specific to *MLH1* and *BRAF* (V600E) status and found that *MLH1* hypermethylation was always accompanied by *BRAF* mutations in both MSI-H1 and MSI-H2, indicating that MSI-H1 did not correspond to Lynch syndrome, because *MLH1* deficiency and *BRAF* mutations rarely occur simultaneously in this disorder. Additionally, consistent with previous findings, a mutual exclusivity was observed between *BRAF* and *KRAS* mutations (Fig. 1b).

In addition, there was no significant association between MSI-H subtypes and tumor locations, sex or American Joint Committee on Cancer (AJCC) stages according to Fisher’s exact test (Fig. 1b). Both subtypes were enriched in stage II/III (75%) and right-sided colon (80%). However, we found that MSI-H1 was enriched in *POLE* mutations (55% in MSI-H1 vs. 30% in MSI-H2). Interestingly, a lower *BRAF* mutational frequency was observed in MSI-H1 (36%) than in MSI-H2 (56%), while no difference was found in the *KRAS* status. This was also the case in GSE39582, with a *BRAF* mutational frequency of 35% and 43% for MSI-H1 and MSI-H2, respectively. However, approximately two-fold higher chromosomal instability was observed in MSI-H1 than in MSI-H2 (50% vs. 29%, GSE39582).

### The tumor immune regulatory network was disrupted in MSI-H1 subtype

**Fig. 2:**
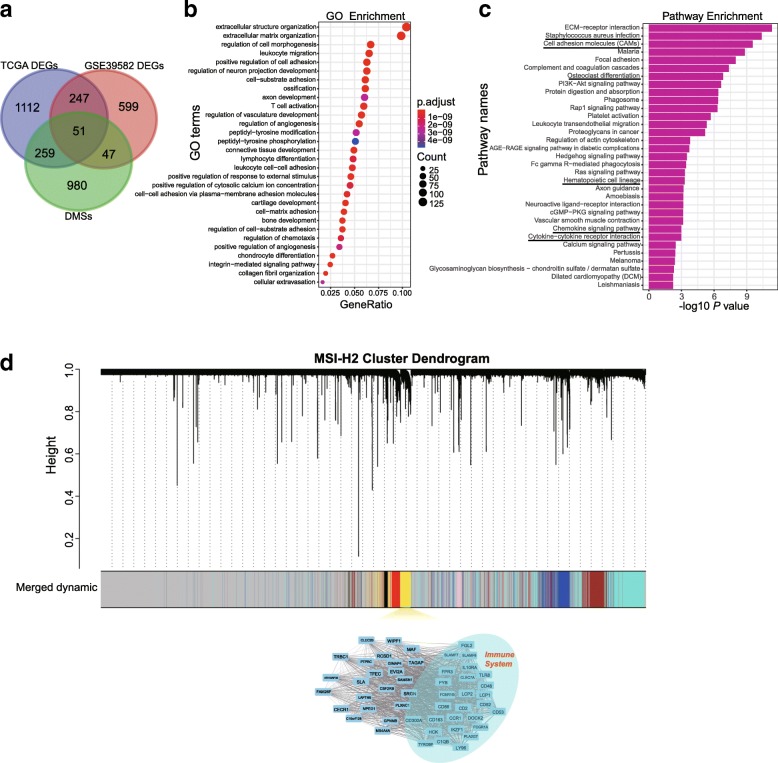
Characterization of functional enrichment of DEGs reside in the MSI-H subtypes. **a**, Venn diagram displaying the number of genes that overlapped within DEGs and DMSs identified in TCGA and GSE39582 datasets. **b**, GO enrichment of up-regulated DEGs found in MSI-H1. **c**, KEGG pathway enrichment of up-regulated DEGs found in MSI-H1. **d**, Coexpression regulatory network identified by WGCNA. Genes that were not coexpressed were assigned to the gray group. Each vertical line corresponds to a gene, and branches are expression modules of highly interconnected groups of genes. In total, seven modules ranging from 146 to 2,939 genes in size were identified in MSI-H2. The yellow module associated with the immune response was selected for visualization

To better understand the molecular difference between MSI-H1 and MSI-H2, it is necessary to determine specific genes that are enriched in each subtype. Differential expression analysis between MSI-H1 and MSI-H2 identified 1,669 and 944 differentially expressed genes (DEGs) in TCGA and GSE39582, respectively, with 298 shared between the two datasets (Fig. 2a, Additional file 6: Table S2). Intriguingly, only 130 genes were down-regulated in MSI-H1. A remarkable 11-fold increase in up-regulated genes found in MSI-H1 inspired us to examine their functional enrichment. GO revealed that these genes were mainly enriched in the immune response, such as regulation of cell adhesion, T cell activation, and lymphocyte differentiation (Fig. 2b). For example, a much higher expression level of CCL2/5, CXCL12, CD86, and CD163 was observed in MSI-H1. KEGG pathway enrichment analysis revealed that MSI-H1 was enriched in genes corresponding to the PI3K-Akt (*P* = 1.7E-7, FDR adjusted), Ras (*P* = 7.8E-4), Rap1 (*P* = 6.2E-7), and Chemokine (*P* = 1.1E-3) signaling pathways (Fig. 2c). We also performed gene set enrichment analyses (GSEA) to decipher the molecular signatures underlying MSI-H1 and MSI-H2 by using the much broader landscape of signatures of hallmark processes collected in the MSigDB database [22]. Notably, epithelial to mesenchymal transition, apical junction, myogenesis, inflammatory response, and KRAS signaling up were enriched in MSI-H1 (Additional file 7: Figure S5 a~d). While E2F targets, MYC targets, G2M checkpoint, glycolysis, mtorc1 signaling, and oxidative phosphorylation had high scores in MSI-H2 (Additional file 7: Figure S5 e~j).

Considering the substantial difference in expression profiles between MSI-H1 and MSI-H2, we speculated that the intrinsic regulatory network also differs to a great extent. To this end, we used weighted correlation network analysis (WGCNA) to determine core gene regulatory modules. Interestingly, seven modules consisting of at least 100 genes were discovered in MSI-H2. Notably, the yellow module represented by CCR1, CD163, CD2, CD52, CD53, and CD86 specific to MSI-H2 was linked to the immune response (Fig. 2d). This observation indicated that the subtle immune regulatory network was lost in MSI-H1, although thousands of immune-related genes were switched on.

### MSI-H subtypes have distinct methylation patterns

**Fig. 3:**
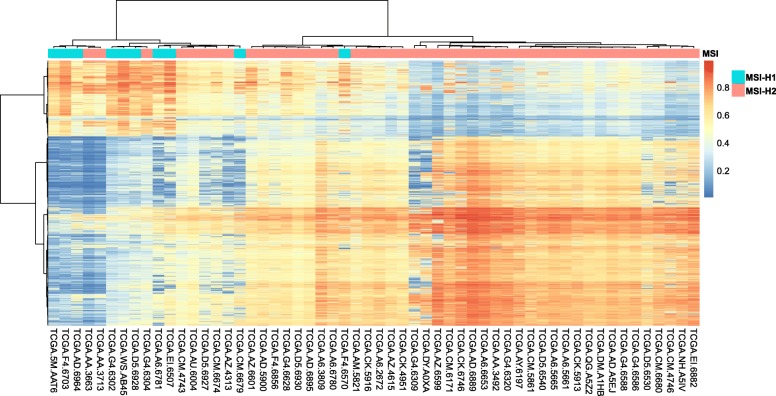
Clustering of 59 MSI-H CRC samples based on DNA methylation profiles. Although the methylation clusters were not identical to the MSI-H subtypes based on expression data, the main trend was preserved

The transcriptome may be too volatile to be affected by some driver mutations. Thus, global DNA methylation pattern was also interrogated between MSI-H subtypes to determine whether the discrepancy between subtypes extended beyond driver mutations. Consistent with the expression pattern, the methylation pattern fell into two main clusters (Fig. 3). A slight global hypomethylated status was observed in MSI-H1. Further in-depth analysis revealed that 3,101 CpGs that covered 1,353 genes were differentially methylated between MSI-H1 and MSI-H2 (Additional file 8: Table S3). Among them, 310 genes overlapped with DEGs identified in TCGA (Fig. 2a). Interestingly, the abovementioned immune-related DEGs were rarely associated with epigenetic regulation (*P* > 0.05, *χ*^2^ test).

In CRC, the dMMR status is strongly associated with the CIMP [21]. Therefore, we wanted to determine whether the CIMP is associated with MSI-H subtypes. Notably, in contrast to chromosomal instability, a much higher frequency of samples with the CIMP was observed in MSI-H2 (67%) than in MSI-H1 (42%, GSE39582), which, to an extent, was consistent the global hypomethylated status of MSI-H1 samples assembled in TCGA. Additionally, no *MLH1*-associated CpGs were differentially methylated; in other words, MSI-H subtypes had little association with *MLH1* methylation status. In addition, the extent of *MLH1* hypermethylation was even higher in MSI-H2 (71%) than in MSI-H1 (55%).

### MSI-H1 has a much poorer prognosis than MSI-H2

**Fig. 4:**
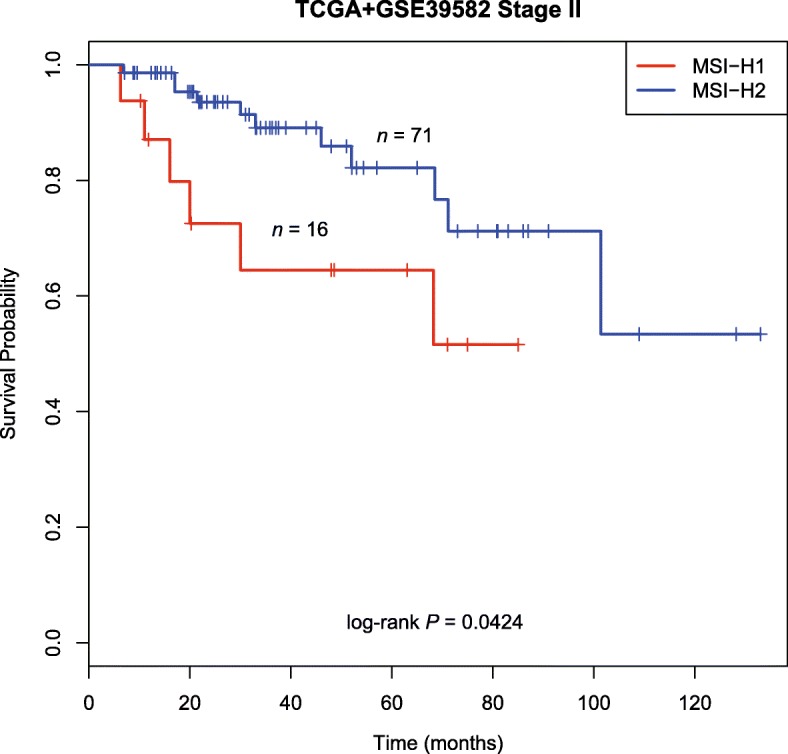
Survival status of stage II MSI-H CRC subtypes. Kaplan-Meier curves showing OS according to MSI-H subtypes. Clearly, a better prognosis was observed for MSI-H2 than for MSI-H1

Previous studies commonly propose that *BRAF* mutations are associated with a poor prognosis [23, 24]. We thus compared overall survival (OS) between MSI-H subtypes using the Cox proportional hazards model. Interestingly, patient survival in MSI-H1 was significantly poorer than that in MSI-H2 (Additional file 9: Figure S6, hazard ratio (HR) = 2.464, 95% confidence interval (CI), 1.13–5.37, *P* = 0.019). The 5-year OS rates were 61% (95% CI, 45 to 84%) and 82% (95% CI, 73 to 93%), respectively. As stage II CRC would be clinically most relevant to MSI-H subtypes, we also compared OS between stage II MSI-H subtypes. The findings held true even after adjusting for stages likely to influence the results (Fig. 4). Nonetheless, it is not known why MSI-H2 CRCs with a higher *BRAF* mutational frequency have a better outcome.

### Worse prognosis of MSI-H1 may be associated with the enrichment of M2 macrophages and PD-L2 expression

**Fig. 5:**
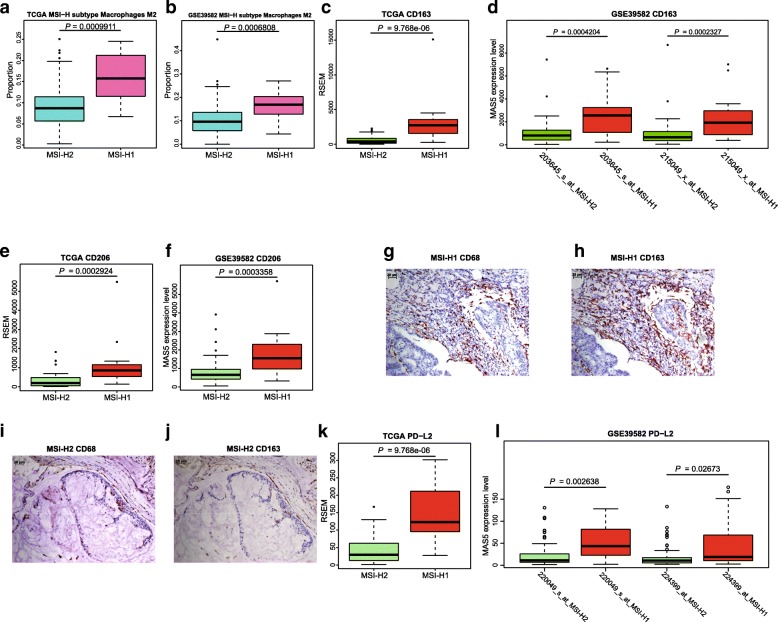
Identification of underlying molecular profiles associated with worse outcomes in MSI-H1. The infiltration of M2 macrophages was more pronounced in MSI-H1 than in MSI-H2 using TCGA (**a**) and GSE39582 (**b**) data. For **c**~**f**, boxplot distribution of *CD163* and *CD206* expression levels using TCGA and GSE39582 data. As for **g**~**j**, representative IHC staining for M2 TAM markers (CD68 and CD163) of serial sections from MSI-H1 and MSI-H2. For **k** and **l**, boxplot distribution of *PD-L2* expression levels using TCGA and GSE39582 data

The lower frequency of *BRAF* mutations in MSI-H1 accompanied by a relatively worse prognosis inspired us to identify the potential tumor microenvironment component underlying this contradictory association. First, we compared the tumor mutational burden (TMB) between MSI-H1 and MSI-H2 because it has a direct impact on the abundance of neoantigens. However, MSI-H1 harbored a similar TMB to MSI-H2 (median mutational frequency ~ 31/million bases, *P* = 0.28, Wilcoxon’s test). Thus, the difference may be related to the participating cell population in the tumor immune microenvironment (TIME), which plays an important role in the poor outcomes in MSI-H1. Deconvolution of 22 lymphocytes in tumor tissue using CIBERSORT revealed a significant enrichment of M2 macrophages in MSI-H1 (Fig. 5a and b, Additional file 10: Table S4, *P* < 0.001). Tumor-associated macrophages (TAMs) of the M2 phenotype are well-known to promote tumor proliferation and are associated with a poor prognosis in different cancer types [25, 26]. CD163 and CD206, two canonical markers of M2 TAMs, had a much higher expression level in MSI-H1 compared to MSI-H2 both in TCGA and GSE39582 (Fig. 5c-f, *P* < 0.001). We then examined the expression of TAM markers (CD68 and CD163) in serial FFPE sections from selected dMMR CRC cases. Notably, we found that CD68 and CD163 had a much higher expression level in MSI-H1 than in MSI-H2 (Fig. 5g-j). This trend also held true for CCL2 and CCL5 (Additional file 11: Figure S7, *P* < 0.01). Tumor-derived CCL2/5 are significant indicators of early relapse and infiltration of TAMs, which contribute to cancer cell proliferation, inflammatory microenvironment of tumors, immune response evasion and angiogenesis [27, 28].

Furthermore, we found that the expression of the immune checkpoint molecule PD-L2 (Programmed cell death 1 ligand 2, also known as PDCD1LG2 or CD273) was significantly higher (> 3-fold) in MSI-H1 than inMSI-H2 (Fig. 5k and l, *P* < 0.001). However, no significant difference was observed in other immune checkpoint molecules such as CTLA-4, PD-1/PD-L1, and LAG-3.

## Discussion

To the best of our knowledge, this is the first systematic subtyping of MSI-H CRC. Both transcriptome and DNA methylome uncovered that two major subtypes were presented in MSI-H CRC, suggesting that MSI-H subtypes was not associated with specific driver mutations. The MSI-H status is well known to confer good prognosis in CRC [2, 29, 30]. Nevertheless, in this study, we found that one-quarter of the MSI-H CRC cases displayed distinct molecular features and poor prognoses. As patients with MSI-H2 CRC had a higher frequency of *BRAF* alterations but a better outcome, we believe that the *BRAF* status is nonsignificant in MSI samples. By evaluating 477 MSI CRC cases, Taieb et al. [31] found that no prognostic role of *BRAF* mutations in patients with MSI CRC. Barras et al. [32] found that CRC cases with *BRAF* mutations can be classified into two subtypes and should not be regarded as having a unique biology that may be exploited for drug targeting. Furthermore, Shimada et al. [33] demonstrated that patients with non-V600E mutant-type cancer had a better OS than patients with V600E mutant-type cancer, which is in line with previous observations [34, 35]. In addition, Laura et al. [36] found that height is a stronger risk factor for CRCs with *BRAF* mutations or MSI, suggesting that molecular pathological epidemiology (MPE) demonstrates the strengths of interdisciplinary integration to study CRC because more and more evidence indicates that diet, smoking, lifestyle, and the microbiome may influence the genome, epigenome, transcriptome, proteome, and metabolome of tumor [37]. Thus, more careful attention should be paid in the treatment of heterogeneous MSI-H population.

Due to defects in DNA MMR, MSI-H tumors have a much higher rate of nonsynonymous mutations, leading to an increased number of neoepitopes and cytotoxic tumor-infiltrating lymphocytes (CTLs). This is the reason why patients with MSI-H CRC generally have more favorable outcomes with immune checkpoint-blockade treatment than patients with microsatellite instability low (MSI-L) or microsatellite stable (MSS) CRC [38]. However, this phenomenon alone can hardly explain the worse survival in MSI-H1 because these tumors also possess huge somatic mutations. In fact, unlike in early-stage disease, MSI is even linked to poorer survival in metastatic CRC [39]. It is tempting to believe that some underlying differences exist in the TIME. Bernhard et al. [40] found that immunoscore was superior to microsatellite instability in predicting patients’ disease-specific recurrence and survival. Coexpression subnetwork construction suggested that the immune regulatory equilibrium in MSI-H1 is off kilter. Further deconvolution of immune infiltrates revealed the enrichment of immunosuppressive M2 TAMs in MSI-H1. M2 TAMs are good targets for anticancer therapy through either ablation or redifferentiation away from protumoral towards antitumoral states because they express canonical markers [41, 42].

PD-L2, which resembles PD-L1, the other ligand of PD-1, is an inhibitor of effector T cells, had a much higher expression in MSI-H1 than in MSI-H2. Overexpression of PD-L2 is associated with poor survival in CRC [43]. Although PD-L2 expression is generally correlated with the expression of PD-L1, PD-L2 is also present in the absence of PD-L1 in subsets of tumor samples [44]. The response rates of a clinical trial including 172 pembrolizumab-treated head and neck squamous cell carcinoma (HNSCC) patients with recurrent or metastatic disease were 23.0% (95% CI, 16.0–31.4) and 26.6% (95% CI, 18.0–36.7) in PD-L1- and PD-L2- positive patients, respectively. These were both much higher than the response rates in the PD-L1- and PD-L2-negative patients (5.9%; 95% CI, 0.1–28.7). Additionally, the overall response rate (ORR) was greatest in patients who were positive for both PD-L1 and PD-L2 and was 2-fold higher (27.5%; 95% CI, 18.6–37.8) than that in patients whose tumors were positive only for PD-L1 (11.4%; 95% CI, 3.2–26.7) [44]. Thus, nominate PD-L2 as a potential novel therapeutic target may be more effective in MSI-H1 CRC.

## Conclusions

In summary, our study showed that not all MSI-H CRC cases share the same molecular characteristics and clinical outcomes. M2 macrophages and PD-L2 reside in the TIME may counteract the prognostic benefit offered by the large amount of neoantigens produced by dMMR tumors. Finally, the heterogeneity in MSI-H tumors indicates that PD-1/PD-L1 does not fit all and that more clinical measures should be addressed for selected patients to improve survival.

## Additional files

Additional file 1: **Table S1.** Clinical information of TCGA and GSE39582 samples used in this study. (XLSX 43 kb)

Additional file 2: **Figure S1.** Clustering of 77 MSI-H CRCs in GSE39582 by NMF. Correlation matrix heatmaps correspond to rank 2 to 6. (PDF 830 kb)

Additional file 3: **Figure S2.** MSI-H CRC patients (GSE39582) fell into two gene expression-based subtypes. (PDF 2907 kb)

Additional file 4: **Figure S3.** Clustering of 48 stage II MSI-H CRCs in TCGA by NMF. Correlation matrix heatmaps correspond to rank 2 to 6. (PDF 488 kb)

Additional file 5: **Figure S4.** Clustering of 53 stage II MSI-H CRCs in GSE39582 by NMF. Correlation matrix heatmaps correspond to rank 2 to 6. (PDF 557 kb)

Additional file 6: **Table S2.** DEGs identified between MSI-H1 and H2 for TCGA and GSE39582. (XLSX 359 kb)

Additional file 7: **Figure S5.** GSEA analysis revealed functional enrichment differences between MSI-H1 and MSI-H2. GSEA was performed for MSI-H CRCs using the hallmark gene signatures collected from MSigDB. (PPTX 276 kb)

Additional file 8: **Table S3.** Differentially methylated sites between MSI-H1 and MSI-H2 identified in this study. (XLSX 251 kb)

Additional file 9: **Figure S6.** Survival status of MSI-H CRC subtypes. (PDF 175 kb)

Additional file 10: **Table S4.** Deconvolution of TCGA and GSE39582 TIL populations by CIBERSORT. (XLSX 52 kb)

Additional file 11: **Figure S7.** Boxplot distribution of *CCL2* and *CCL5* expression level between MSI-H1 and MSI-H2 by using TCGA and GSE39582 data. (PDF 412 kb)

## Data Availability

The datasets generated and/or analysed during the current study are available in the TCGA repository, https://cancergenome.nih.gov/.

## Abbreviations

CIMP: CpG island methylator phenotype
CMS: Consensus molecular subtype
CRC: Colorectal cancer
CTLs: Cytotoxic tumor-infiltrating lymphocytes
DEGs: Differentially expressed genes
DFS: Disease-free survival
DMSs: Differentially methylated sites
FDR: False discovery rate
GEO: Gene Expression Omnibus
GO: Gene Ontology
GSEA: Gene set enrichment analyses
HNSCC: Head and neck squamous cell carcinoma
HR: Hazard ratio
KEGG: Kyoto Encyclopedia of Genes and Genomes
Mb: Million bases
MMR: Mismatch repair system
MSI-H: Microsatellite instability-high
MSI-H1: MSI-H group 1
MSI-H2: MSI-H group 2
MSI-L: Microsatellite instability low
MSS: Microsatellite stable
NMF: Nonnegative matrix factorization
OS: Overall survival
QN: Quantile normalization
TAMs: Tumor-associated macrophages
TCGA: The Cancer Genome Atlas
TIME: Tumor immune microenvironment
TMB: Tumor mutational burden
