# Supplementary figures and images for: Subtyping of microsatellite instability-high colorectal cancer
Source: Cell Commun Signal. 2019 Jul 22;17:79. doi: 10.1186/s12964-019-0397-4 (PMC6647262; doi:10.1186/s12964-019-0397-4)

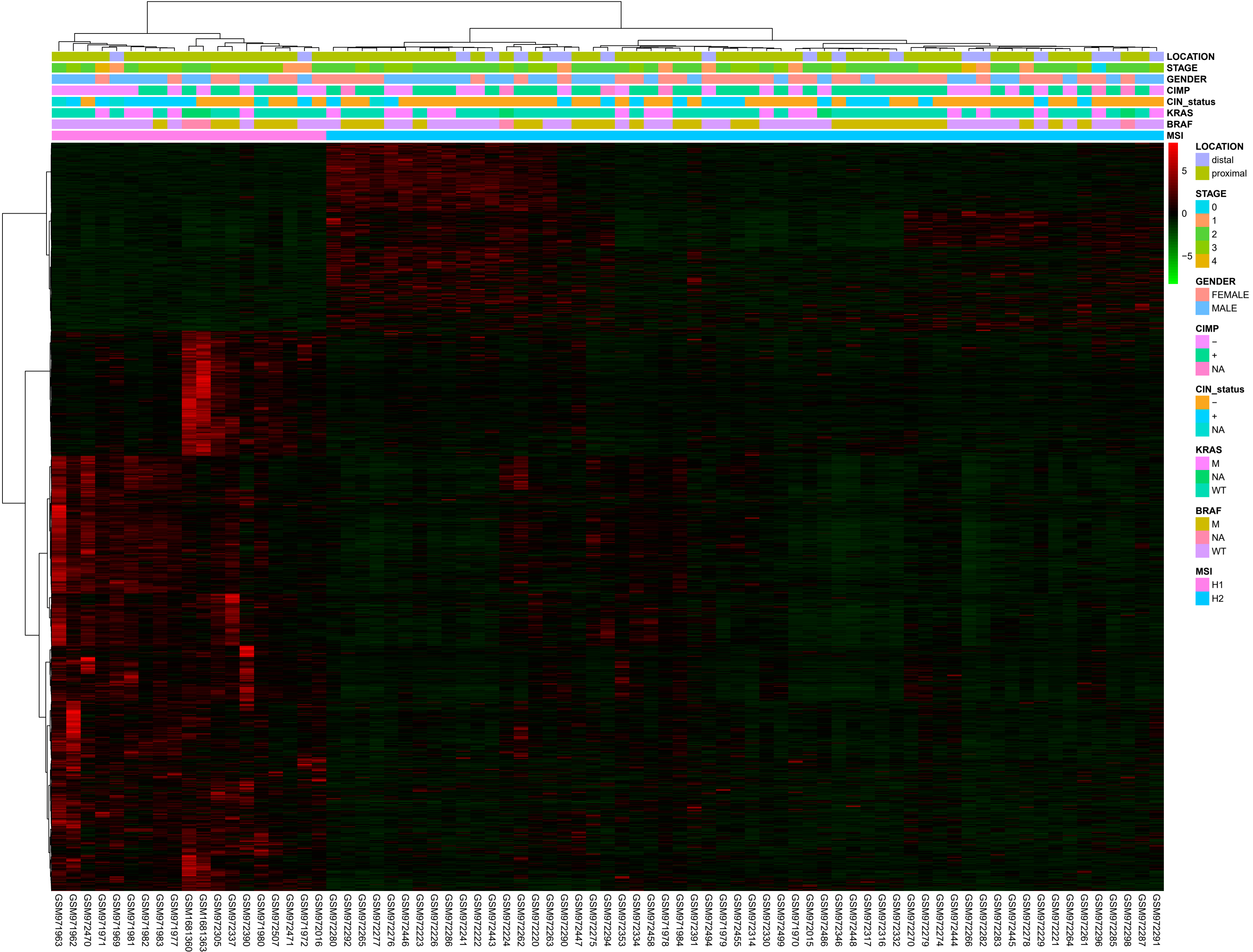

Supplement: Supplementary file 3 — Figure S2. MSI-H CRC patients (GSE39582) fell into two gene expression-based subtypes. (PDF 2907 kb) [file 12964_2019_397_MOESM3_ESM.pdf]
