# Supplementary material for: Subtyping of microsatellite instability-high colorectal cancer
Source: Cell Commun Signal. 2019 Jul 22;17:79. doi: 10.1186/s12964-019-0397-4 (PMC6647262; doi:10.1186/s12964-019-0397-4)
Supplement: Supplementary file 5 — Figure S4. Clustering of 53 stage II MSI-H CRCs in GSE39582 by NMF. Correlation matrix heatmaps correspond to rank 2 to 6. (PDF 557 kb) [file 12964_2019_397_MOESM5_ESM.pdf]

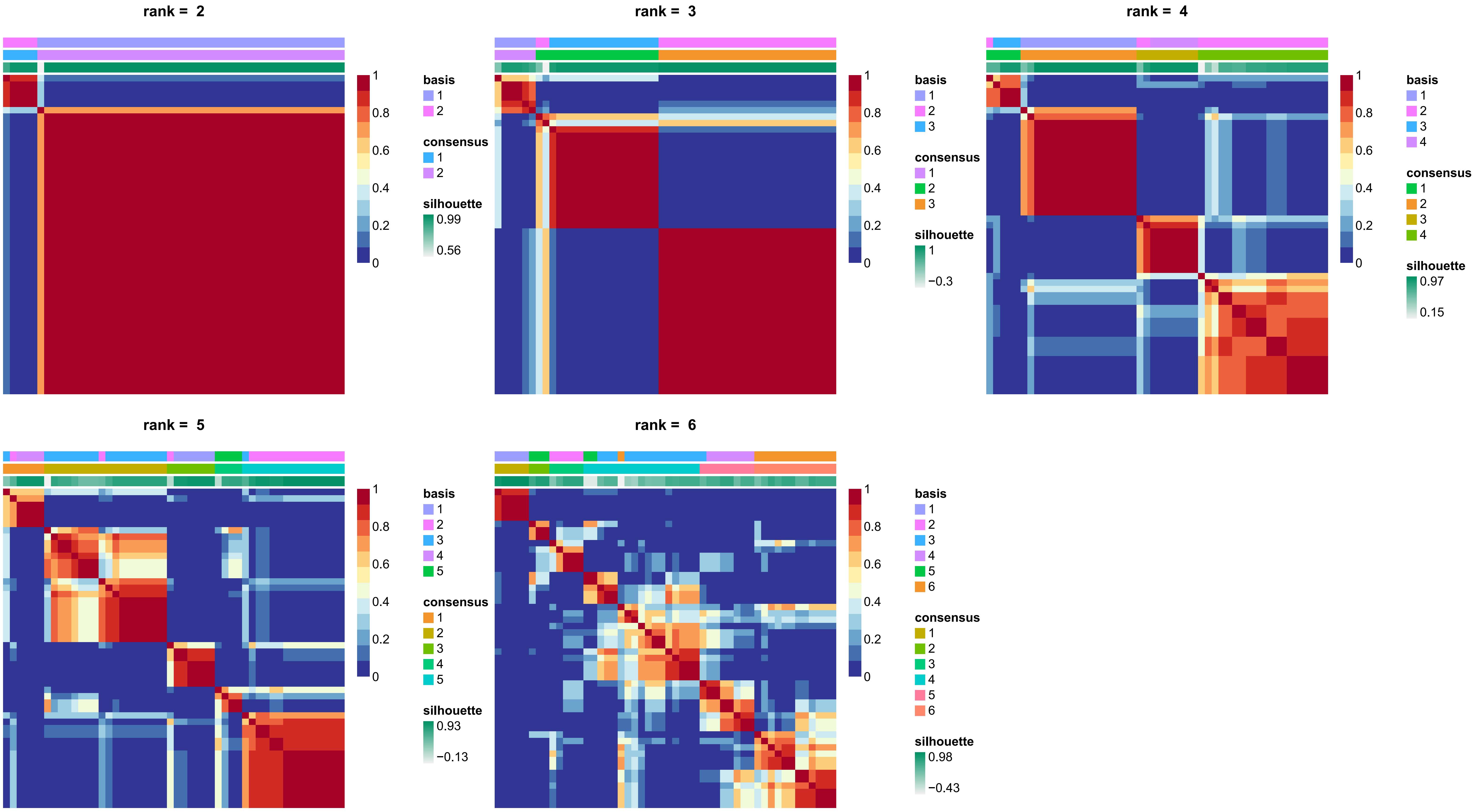

Figure S4 Clustering of 53 stage II MSI-H CRCs in GSE39582 by NMF. Correlation matrix heatmaps correspond to rank 2 to 6.
