# Supplementary material for: Subtyping of microsatellite instability-high colorectal cancer
Source: Cell Commun Signal. 2019 Jul 22;17:79. doi: 10.1186/s12964-019-0397-4 (PMC6647262; doi:10.1186/s12964-019-0397-4)
Supplement: Supplementary file 7 — Figure S5. GSEA analysis revealed functional enrichment differences between MSI-H1 and MSI-H2. GSEA was performed for MSI-H CRCs using the hallmark gene signatures collected from MSigDB. (PPTX 276 kb) [file 12964_2019_397_MOESM7_ESM.pptx]

## Slide 1
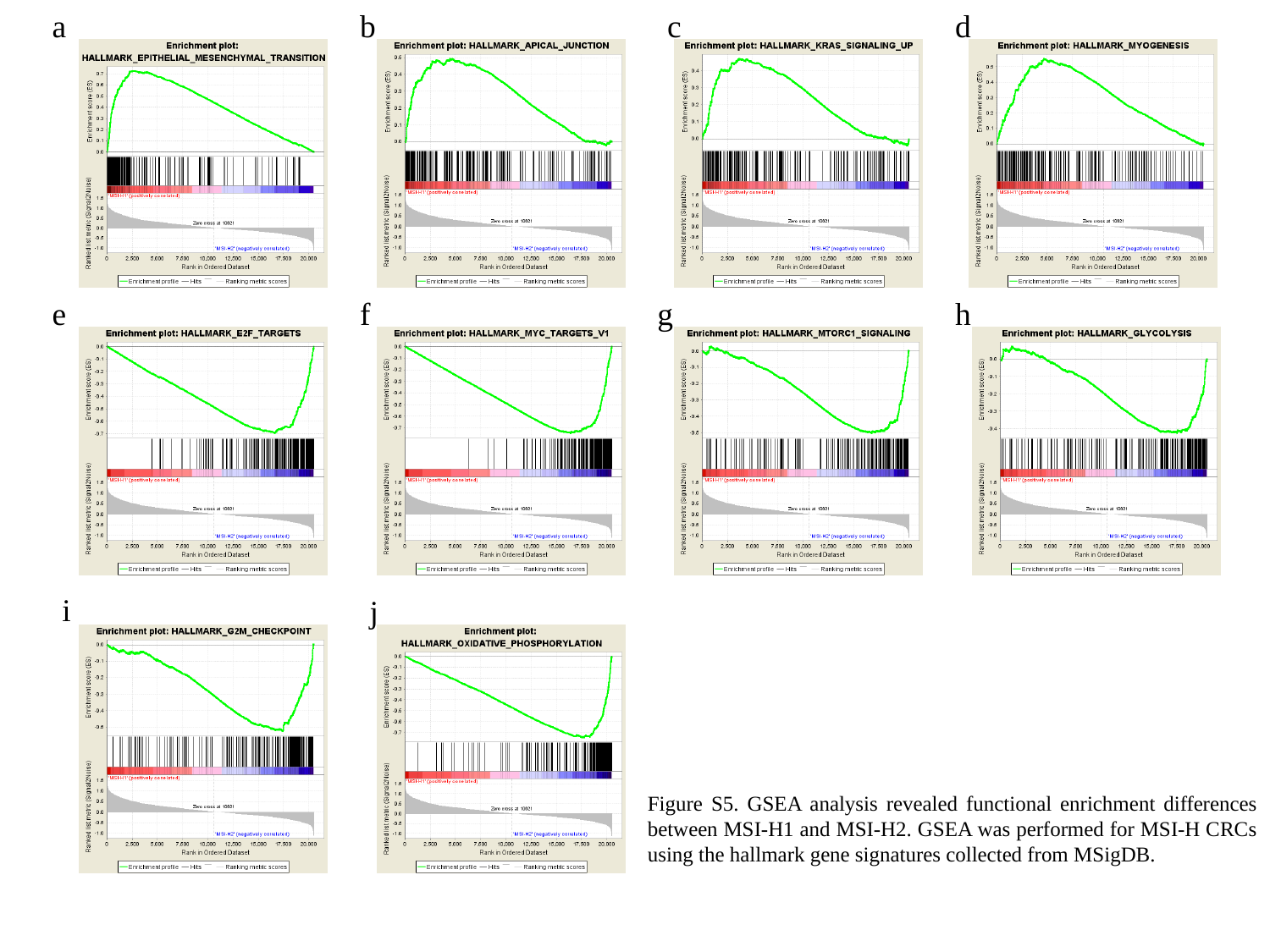

a
b
c
d
e
f
g
h
i
j
Figure S5. GSEA analysis revealed functional enrichment differences between MSI-H1 and MSI-H2. GSEA was performed for MSI-H CRCs using the hallmark gene signatures collected from MSigDB.
