# Supplementary material for: Subtyping of microsatellite instability-high colorectal cancer
Source: Cell Commun Signal. 2019 Jul 22;17:79. doi: 10.1186/s12964-019-0397-4 (PMC6647262; doi:10.1186/s12964-019-0397-4)
Supplement: Supplementary file 9 — Figure S6. Survival status of MSI-H CRC subtypes. (PDF 175 kb) [file 12964_2019_397_MOESM9_ESM.pdf]

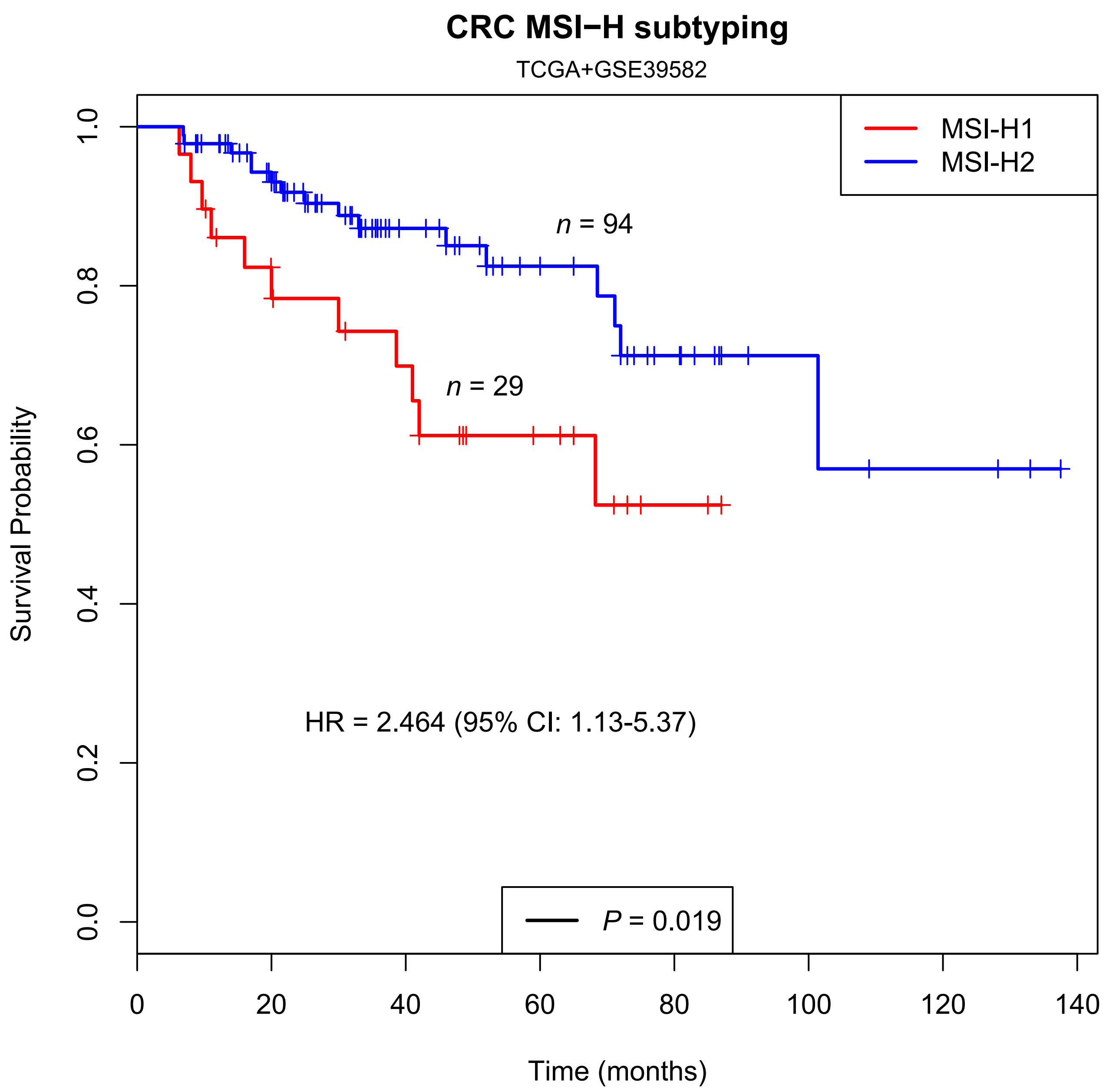

Figure S6 Survival status of MSI-H CRC subtypes. Kaplan-Meier curves showing OS according to MSI-H subtypes. Clearly, a better prognosis was observed for MSI-H2 than for MSI-H1. The HR shown on the graph is derived from univariable Cox proportional hazards model testing. Abbreviations: CI, confidence interval; HR, hazard ratio; ***P***, P-value.
