# Supplementary material for: Subtyping of microsatellite instability-high colorectal cancer
Source: Cell Commun Signal. 2019 Jul 22;17:79. doi: 10.1186/s12964-019-0397-4 (PMC6647262; doi:10.1186/s12964-019-0397-4)
Supplement: Supplementary file 11 — Figure S7. Boxplot distribution of CCL2 and CCL5 expression level between MSI-H1 and MSI-H2 by using TCGA and GSE39582 data. (PDF 412 kb) [file 12964_2019_397_MOESM11_ESM.pdf]

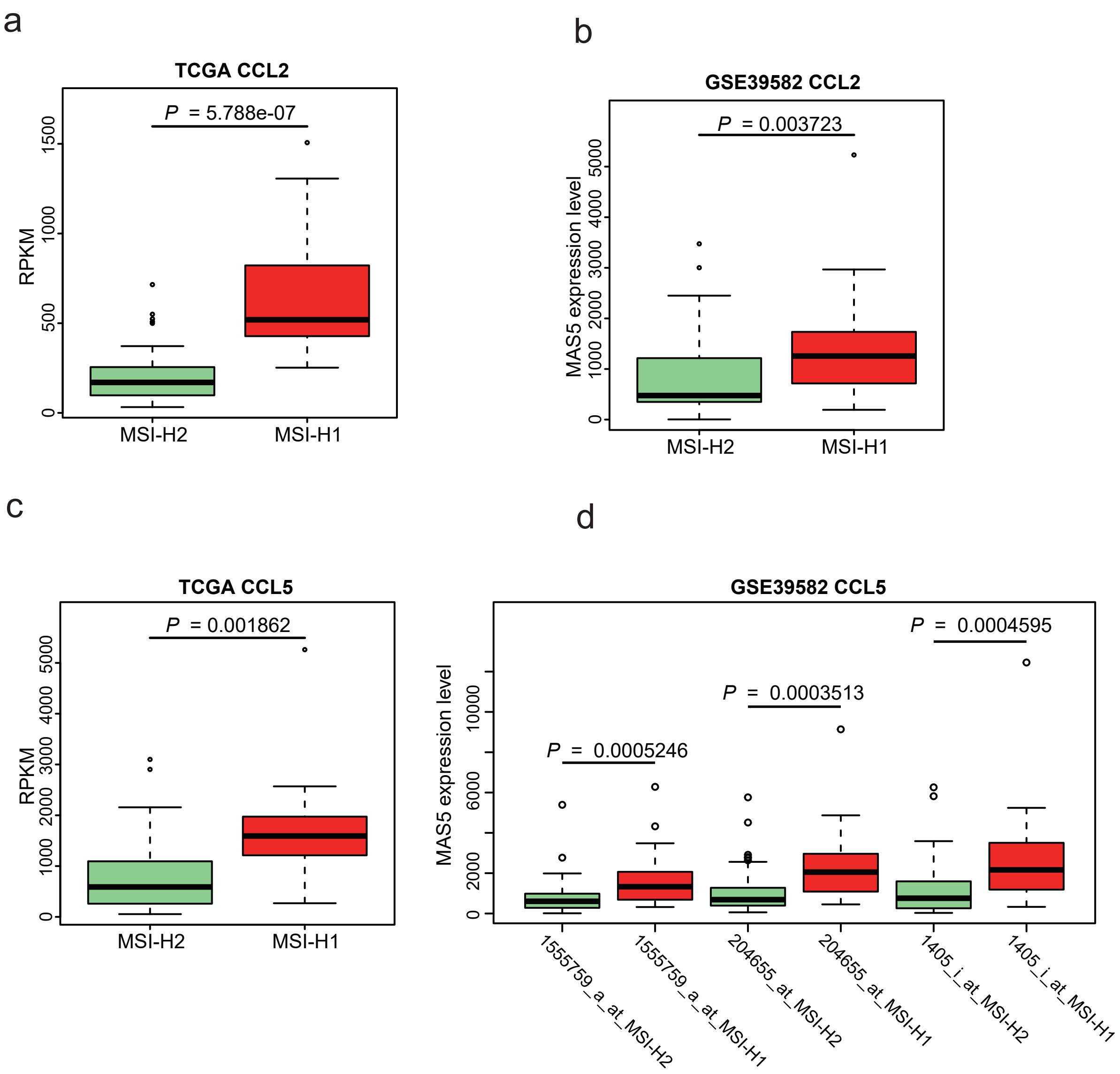

Figure S7 Boxplot distribution of *CCL2* and *CCL5* expression level between MSI-H1 and MSI-H2 by using TCGA and GSE39582 data.
